# Bone as a site of insulin resistance in type 2 diabetes

**DOI:** 10.1186/1753-6561-6-S3-P74

**Published:** 2012-06-27

**Authors:** Jianwen Wei, Gerard Karsenty

**Affiliations:** 1 Department of Genetics & Development, College of Physicians and Surgeons, Columbia University, New York, NY 10032, USA

## Background

Type 2 diabetes is characterized by insulin resistance in various insulin target cells, such as hepatocytes, myoblasts and adipocytes [1]. The recent realization that the osteoblast is an insulin target cell involved in the control of whole-body glucose homeostasis [2] suggests the possibility that impaired insulin signaling in osteoblasts contributes to the development of type 2 diabetes.

## Materials and methods

All mice were fed a high fat diet (60%kcal%fat) for 8 weeks to induce diabetes. To examine insulin signaling in bones, phosphorylation levels of InsR at Tyr1150/1151, AKT at Thr308 and Ser473 and GSK-3-β at Ser9 were determined by Western blots. Glucose metabolism and insulin sensitivity were determined by Glucose tolerance test (GTT) and Insulin tolerance tests (ITT). Serum levels of insulin, osteocalcin and bone resorption marker Ctx were measured by ELISA.

## Results

To determine whether bone is a site of insulin resistance, we first examined insulin signaling in bone tissues of WT mice fed a high fat diet (HFD) and observed that levels of insulin receptor, insulin-induced phosphorylation of insulin receptor at Tyr1150/1151, of AKT at Thr308 and Ser473, and of GSK-3-β at Ser9, were markedly decreased. More importantly, several insulin-dependent functions taking place in osteoblasts were disrupted in mice rendered diabetics through HFD: **(1)** bone resorption was decreased as reflected by reduced level of serum Ctx. **(2)** expression of *Opg*, an insulin target gene in osteoblast, was increased by 2 fold compared to control mice. **(3)** circulating levels of active osteocalcin (Glu13-Ocn) was decreased in HFD-fed mice. In addition, ex vivo treatment of primary osteoblasts with palmitate, one of the most abundant dietary saturated fatty acid causing insulin resistance in various insulin target cells, also significantly impaired insulin signaling, as shown by decreased levels of insulin receptor and insulin stimulated phospho-AKT. Lastly, to test If insulin resistance in osteoblasts contribute to the systematic insulin resistance, we used loss-of (*InsR_osb_*+*/-*) and gain-of function (*α1*(*I*)*Col-InsR*) models of insulin signaling in osteoblasts*.* Although showing normal glucose metabolism when fed on normal diet, *InsR_osb_*+*/-* mice developed more severe insulin resistance in comparison to control mice fed the same HFD as evidenced by higher levels of blood glucose and serum insulin, decreased ability to clear glucose in GTT and more resistance to insulin in ITT. Conversely, *α1*(*I*)*Col-InsR* mice fed on HFD were less hyperglycemic, less hyperinsulinemic, more glucose tolerance and more insulin sensitive than control mice.

## Conclusions

Taken together, this study provided evidence that insulin resistance takes place in bones in diet-induced type 2 diabetes model and insulin resistance in osteoblasts contributes to the development of systematic insulin resistance in type 2 diabetes.
